# An optimized protocol for the preparation of oxygen-evolving thylakoid membranes from *Cyclotella meneghiniana* provides a tool for the investigation of diatom plastidic electron transport

**DOI:** 10.1186/s12870-017-1154-8

**Published:** 2017-11-25

**Authors:** Marcel Kansy, Alexandra Gurowietz, Christian Wilhelm, Reimund Goss

**Affiliations:** Department of Plant Physiology, Institute of Biology, University Leipzig, Johannisallee 21-23, D-04103 Leipzig, Germany

**Keywords:** Diatom, NPQ, Oxygen evolution, Photosynthetic electron transport, Proton gradient, Thylakoid membrane, Xanthophyll cycle

## Abstract

**Background:**

The preparation of functional thylakoid membranes from diatoms with a silica cell wall is still a largely unsolved challenge. Therefore, an optimized protocol for the isolation of oxygen evolving thylakoid membranes of the centric diatom *Cyclotella meneghiniana* has been developed. The buffer used for the disruption of the cells was supplemented with polyethylene glycol based on its stabilizing effect on plastidic membranes. Disruption of the silica cell walls was performed in a French Pressure cell and subsequent linear sorbitol density gradient centrifugation was used to isolate the thylakoid membrane fraction.

**Results:**

Spectroscopic characterization of the thylakoids by absorption and 77 K fluorescence spectroscopy showed that the photosynthetic pigment protein complexes in the isolated thylakoid membranes were intact. This was supported by oxygen evolution measurements which demonstrated high electron transport rates in the presence of the artificial electron acceptor DCQB. High photosynthetic activity of photosystem II was corroborated by the results of fast fluorescence induction measurements. In addition to PSII and linear electron transport, indications for a chlororespiratory electron transport were observed in the isolated thylakoid membranes. Photosynthetic electron transport also resulted in the establishment of a proton gradient as evidenced by the quenching of 9-amino-acridine fluorescence. Because of their ability to build-up a light-driven proton gradient, de-epoxidation of diadinoxanthin to diatoxanthin and diatoxanthin-dependent non-photochemical quenching of chlorophyll fluorescence could be observed for the first time in isolated thylakoid membranes of diatoms. However, the ∆pH, diadinoxanthin de-epoxidation and diatoxanthin-dependent NPQ were weak compared to intact diatom cells or isolated thylakoids of higher plants.

**Conclusions:**

The present protocol resulted in thylakoids with a high electron transport capacity. These thylakoids can thus be used for experiments addressing various aspects of the photosynthetic electron transport by, e.g., employing artificial electron donors and acceptors which do not penetrate the diatom cell wall. In addition, the present isolation protocol yields diatom thylakoids with the potential for xanthophyll cycle and non-photochemical quenching measurements. However, the preparation has to be further refined before these important topics can be addressed systematically.

**Electronic supplementary material:**

The online version of this article (10.1186/s12870-017-1154-8) contains supplementary material, which is available to authorized users.

## Background

Diatoms (Bacillariophyceae) are eukaryotic, unicellular algae, which are found in both marine and freshwater ecosystems. These various ecological habitats are characterized by sometimes drastic differences in their abiotic environment [28]. The ecophysiological role of photosynthetic diatoms is of huge importance, since they contribute significantly to the total CO_2_ fixation and biomass production in the oceans, being a key regulatory component in global carbon cycling [1]. Diatoms phylogenetically originate from a secondary endocytobiotic event, described as engulfment of a red alga-like photoautotroph by a heterotrophic eukaryote during evolution [46]. As a result, the diatom chloroplast is characterized by a more complicated plastid structure in comparison to chloroplasts of the green lineage. Although varieties in chloroplast morphology within the group of diatoms have been reported, the general chloroplast structure shows certain typical features [3]. Most notably, as a result of secondary endosymbiosis, the diatom chloroplast stroma is surrounded by four membranes, i.e. a double-layered chloroplast envelope that, in turn, is encircled by a double-layered so-called chloroplast endoplasmatic reticulum. Another unique feature is the organization of the thylakoid membranes. In contrast to the grana and stroma lamellae, which are typical for chloroplasts of the green lineage [5], the diatom thylakoids form regular stacks of three membranes. Besides the differences in the structural organisation, variances in the biochemical composition of the diatom thylakoid membrane in comparison to higher plants and green algae have been reported. These differences are found in the membrane proteome [19], the lipid composition of the thylakoid membrane matrix [33] and also the pigment composition of the membrane-embedded photosynthetic protein complexes [28]. Together, the pronounced differences in both the structural arrangement and the biochemical composition of diatom chloroplasts and thylakoids clearly distinguishes them from green algae and higher plants.

In order to cope with a fast changing abiotic environment, excitation energy transfer and electron transport mechanisms are highly flexible in photosynthetic organisms. This is an important feature to overcome imbalances in the ratio between energy absorption and utilization. Overexcitation of the photosynthetic apparatus could lead to the production of reactive oxygen species which, in turn, would severely limit photosynthetic electron transport rates and subsequently cell growth. Taking into account the natural environment of diatoms, their photosynthetic apparatus has to provide an especially high degree of flexibility in the adaptation to rapidly changing light conditions. To investigate and understand the mechanisms of diatom photosynthesis in close detail, measurements with intact, native cells are often insufficient. Manipulation of isolated chloroplasts and/or thylakoid membranes with, e.g., different electron acceptors or uncouplers of the proton gradient, which have been performed in countless studies on higher plant chloroplasts and thylakoid membranes, are needed. Insight into important partial reactions of the overall photosynthetic process will then provide the means to obtain a new and improved understanding of diatom photosynthesis in general.

In contrast to higher plants and green algae, only few reports exist about the isolation of intact diatom chloroplasts and/or thylakoids. Despite the low number of preparation protocols, all of these studies demonstrate that the biggest obstacle for the preparation of intact plastids and photosynthetic membranes from diatoms is represented by the silica frustule. The frustule is constructed from amorphous silica particles forming a very stable shell, which mainly protects the cell against mechanical stress [1]. Consequently, opening the diatom cells to get access to the organelles demands strong shearing forces, which are high enough to potentially destroy the functionality of the released intracellular organelles or macro-domain structures. Even more, the integrity of membranes, including the chloroplast envelope and thylakoid membranes, and the membrane integral and membrane associated proteins, such as the oxygen evolving complex of PSII, can be severely damaged. The first protocol on the topic of thylakoid isolation from diatom cells was published by Murata et al. [38] for the pennate diatom *Phaeodactylum tricornutum*. However, in contrast to *Cyclotella meneghiniana* that was used in the present study, *P. tricornutum*, does not contain a silica cell wall. Later, three further protocols for the preparation of intact diatom plastids and thylakoids were presented by Wittpoth et al. [55], Martinson et al. [36] and Nagao et al. [39]. These protocols led to the successful preparation of photosynthetically active thylakoids or even chloroplasts of the diatom species *Odontella sinensis*, *Coscinodiscus granii*, *Cylindrotheca fusiformis*, and *Chaetoceros gracilis*, respectively. With the exception of *P. tricornutum* only a limited number of physiological studies exist for the diatom species used for the isolation procedures that yielded intact and active membranes. *Cyclotella meneghiniana*, on the other hand, is a well-studied diatom and numerous reports about the structure and function of the fucoxanthin chlorophyll proteins (FCP, for a recent review see [6]) or the nature of non-photochemical fluorescence quenching (NPQ, reviewed in [16]) have been presented. In addition, the physiology of *C. meneghiniana*, including the plastidic electron transport, shows differences to that of other diatoms, e.g. *P. tricornutum* (for a review see [15]).

The present paper describes an optimized protocol for the isolation of intact thylakoid membranes from *C. meneghiniana*, followed by a characterization of the structure and function of the purified membranes. In addition, the potential of the oxygen-evolving thylakoids for studies of specific partial reactions of the photosynthetic electron transport or the mechanism of NPQ was investigated. The investigations included Hill reaction measurements, determination of the light-driven proton gradient by fluorescence quenching of the ΔpH sensitive fluorophore 9-amino-acridine, as well as first NPQ and diadinoxanthin (DD) de-epoxidation experiments. To determine the potential of the isolated membranes for these fields of investigation is especially interesting because in diatom thylakoid membranes a strong chlororespiratory electron transport can be observed, both during longer dark periods and high light illumination [10, 18, 25, 26]. In addition, diatoms show a pronounced cyclic electron transport around PSII [30] and NPQ seems to be regulated differently compared to higher plants (for a review see [16]).

## Methods

### Culture growth conditions

The diatom *C. meneghiniana* (strain 1020-1a; SAG Culture Collection of Algae at Göttingen University) was grown as air-lift culture in 2 L of f/2 medium supplemented with vitamins according to Guillard [20] with the following modifications: The salt (Tropic Marin, Dr. Biener GmbH, Germany) content was reduced by 50% and the amount of silica was doubled. Cultivation was done at 20 °C with a light-dark regime of 14/10 h under low light conditions of an ambient light intensity of 40 μmol m^−2^ s^−1^ (Lumilux Cool White L36 W/840, Osram, Germany).

### Isolation and purification of thylakoid membranes

**Fig. 1:**
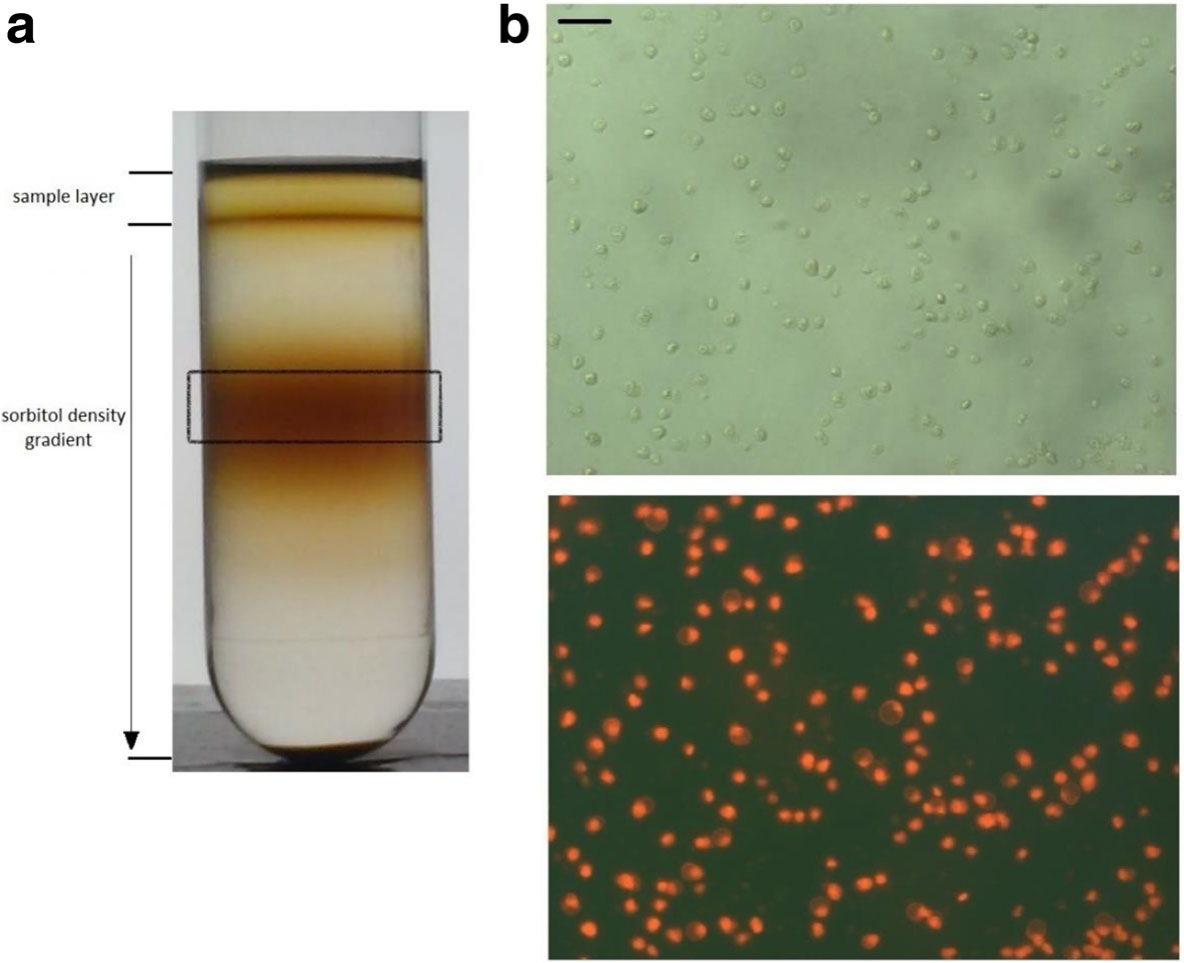
**a** Linear gradient from 2 to 3.3 M sorbitol, (**b**) brightfield (upper panel) and fluorescence (lower panel) microscopic images of the fraction representing the isolated thylakoids of *C. meneghinana* (both images show the same section, bar represents 2 μm). 2 mL of the FrenchPress extract (approx. 0.8–1 mg Chl mL^−1^) were loaded onto the sorbitol gradient. For further details concerning the centrifugation step see the Methods section

For thylakoid preparation seven to 9 day old cultures with a chlorophyll (Chl) concentration of around 4–5 mg L^−1^ were harvested by centrifugation with 1.500 g for 10 min at a temperature of 4 °C (Varifuge 3.0 R, Heraeus, Germany). The resulting cell pellet was resuspended in 10 mL ice cold isolation buffer (0.1% BSA *w*/*v*; 400 mM glycin betaine; 5 mM Na-EDTA; 5 mM ε-aminocaproic acid; 10 mM KCl; 25 mM MOPS-KOH pH 6.7 RT) and afterwards centrifuged for 4 min at 4 °C with 600 g (Allegra 64R, Beckman, USA). The final cell pellet was again resuspended in isolation buffer, supplemented with 40% polyethylene glycol (PEG) 4000 (*w*/*v*), and immediately passed through a pre-cooled French Pressure cell (Thermo Spectronic, UK) with a chamber pressure of 1.000 psi. To purify the thylakoid membranes 2 mL of the resulting cell extract were directly loaded on top of a linear sorbitol density gradient ranging from 2 to 3.3 M sorbitol (w/v; dissolved in the buffer described above). The sorbitol gradients were centrifuged in an Optima C-90 K ultracentrifuge equipped with a SW-28 rotor (Beckman, USA) at 16.000 g and 4 °C for 30 min. After centrifugation, the main brown coloured band (see Fig. 1a) containing the thylakoid membranes was harvested with a syringe. The collected sample was diluted with a similar volume of isolation buffer and centrifuged at 10.000 g and 4 °C for 10 min (Allegra 64R, Beckman, USA) to pellet the thylakoid fraction. One part of the pellet was resuspended in a shock medium (10 mM KCl; 10 mM MOPS-KOH pH 7.0 RT) and remaining chloroplasts were osmotically broken by a 2 min incubation. After the incubation in shock medium the thylakoids were again centrifuged at 10.000 g and 4 °C for 10 min.

The resulting pellet was resuspended in a small volume of isolation buffer (without PEG) and stored on ice in the dark. The chlorophyll concentration of the isolated thylakoid membranes was determined in 90% acetone according to Jeffrey and Humphrey [24].

### Determination of the protein composition of the thylakoids by blue-native (BN)- and SDS-PAGE

A volume of the thylakoid sample containing 100 μg Chl was centrifuged for 10 min at 10.000 g and 4 °C (Allegra 64R, Beckman, USA). The faint coloured supernatant was discarded and the pellet was resuspended in 100 μL solubilisation buffer as described in Järvi et al. [23], with a final n-dodecyl β-D-maltoside (Roth, Germany) concentration of 2% (*w*/*v*). The sample was gently stirred for 20 min in the dark on ice. Following solubilisation the sample was again centrifuged for 10 min at 10.000 g, 4 °C and 8 μL of the supernatant were supplemented with 2 μL of a 5 x BN-PAGE loading buffer [23]. BN-PAGE was performed as described in Schägger and von Jagow [47] in self-cast gradient gels with an acrylamide concentration from 3 to 15% and a 3% stacking gel (BioRad Mini-PROTEAN Tetra Cell system; acrylamide stock solution 30% acrylamide, 0.8% bisacrylamide, *w*/*v*, Roth, Germany). Different electric field conditions were applied during the separation process as recommended by Järvi et al. [23]. Coomassie staining solution was prepared and used as described by Dyballa and Metzger [9].

The polypeptide composition of the isolated thylakoid membranes was analysed by SDS-PAGE according to Laemmli [29]. The stacking and separation gels were prepared with acrylamide concentrations of 5 and 15%, respectively, from the stock solution described above. Molecular weight marker proteins (test mixtures 4 and 5) were from Serva (Germany). For the electrophoresis thylakoid samples with a Chl content of 1.5 μg were applied to the gel. After electrophoresis the separated protein bands were stained with a colloidal Coomassie solution according to Dyballa and Metzger [9].

### Spectroscopic characterization of isolated thylakoids

Absorption spectra were recorded at room temperature with a Specord 250 spectrophotometer (Zeiss, Germany) in the wavelength range from 350 to 750 nm. A bandpass setting of 1 nm was chosen and the Chl concentration of the thylakoids was adjusted to 2 μg mL^−1^. 77 K fluorescence spectra were measured with a FluoroMax-4 spectrofluorometer (Jobin Yvon Horriba, USA) in a buffer supplemented with 60% glycerol (*v*/v). The Chl concentration used for the 77 K fluorescence spectroscopy was 1 μg mL^−1^. Fluorescence emission spectra were recorded in the wavelength range from 600 to 800 nm. The excitation wavelength was set to 440 nm and the bandpasses for the emission and excitation were set to 2 nm and 5 nm, respectively. Fluorescence excitation spectra were recorded in the wavelength range from 400 to 550 nm. The fixed emission wavelength was selected according to the position of the Chl a emission maximum determined in the previously recorded emission spectra. For the excitation spectra the bandpass settings for the emission and excitation were 5 and 2 nm, respectively. The correction of the excitation spectra was done automatically by calibration with the spectrum of the light source that was recorded during the sample measurements.

### Oxygen evolution measurements

The electron transport rates of the isolated thylakoid membranes were measured as oxygen evolution with a Clark-type oxygen electrode (Hansatech Instruments, UK). The measurements were performed in the isolation buffer of the thylakoid preparation (without PEG) at a temperature of 24 °C. Thylakoids were used with a Chl concentration of 10 μg mL^−1^. 2,6-dichloro-*p*-benzoquinone (DCBQ) or ferricyanide (FeCN) were used as artificial electron acceptors with final concentrations of 0.5 mM and 2 mM, respectively. To uncouple the light-driven proton gradient the reaction buffer was supplemented with 10 mM ammonium chloride (NH_4_Cl). All supplements were added prior to the onset of actinic measuring light at non-actinic ambient light conditions. Following a short period of dark adaptation, the thylakoids were illuminated with actinic white light of an intensity of around 300 μmol m^−2^ s^−1^.

### Fast fluorescence induction measurements

Fast Chl fluorescence induction curves were recorded with a Plant Efficiency Analyser (PEA, Hansatech Instruments, UK) as described in [50] at room temperature. Following dark adaptation of intact cells or isolated thylakoids for 3 min in the measuring cuvette, the fluorescence was induced by a 1 s saturating light flash with an intensity of 3.500 μmol m^−2^ s^−1^. The fluorescence rise was recorded during the time of the flash. For comparison of different samples the curves were normalized to their respective F_m_ and F_0_ values, with F_0_ defined as the fluorescence at the time point of 20 μs. Thylakoid membranes were measured with a Chl concentration of 2 μg mL^−1^ in the absence (control) or presence of the artificial electron acceptors FeCN (2 mM) or DCBQ (0.5 mM).

### Determination of non-photochemical quenching and xanthophyll cycle activity

For the determination of NPQ and the xanthophyll cycle activity the isolated thylakoids were diluted in the isolation buffer of the thylakoid preparation (without PEG) to yield a final Chl concentration of 10 μg mL^−1^. The cosubstrate of the de-epoxidation reaction ascorbate (30 mM) was added before the start of the actinic illumination. To suppress the conversion of DD to diatoxanthin (Dt) the DD de-epoxidase inhibitor dithiothreitol (DTT) was added at a concentration of 3 mM. The thylakoids were illuminated at room temperature in the measuring cuvette (ED 101 US, Walz, Germany) of the PAM fluorometer (PAM 101, Walz, Germany) for 25 min with a light intensity of 1.000 μmol m^−2^ s^−1^. Saturating light pulses of a duration of 1 s and a light intensity of 3.500 μmol m^−2^ s^−1^ were applied every minute. NPQ was calculated as Stern-Volmer Quenching [F_m_/F_m__′_-1] according to Bilger and Björkman [4]. After the end of the actinic illumination the thylakoid pigments were extracted and analysed by HPLC as described in detail by Schaller et al. [48]. Pigment concentrations were calculated according to Lohr and Wilhelm [35]. The de-epoxidation state (DES) of the DD cycle pigment pool was calculated as [Dt/(DD + Dt)].

### Determination of 9-amino-acridine fluorescence quenching

The magnitude of the light-driven proton gradient was determined by 9-amino-acridine fluorescence (9-AAF) quenching according to Schuldiner et al. [49]. 9-AAF was measured with a XE-PAM (Walz, Germany) equipped with a special filter set (FS-9AA, Walz, Germany). Thylakoid suspensions were used with a total Chl concentration of 15 μg mL^−1^, the 9-AAF concentration was 6 μM. FeCN (2 mM) or DCBQ (0.5 mM) were added as artificial electron acceptors. 9-AAF quenching was detected during an illumination with actinic light of an intensity of about 500 μmol m^−2^ s^−1^ and a dark period following the illumination phase. Measurements were performed at room temperature in an ED 101 US cuvette (Walz, Germany) under gentle stirring of the reaction mixture. The magnitude of the ∆pH was calculated as ∆F/F value with F being the 9-AAF before the onset of actinic illumination and ∆F being the difference between the 9-AAF before the illumination and the 9-AAF during the actinic light phase.

## Results and discussion

### Optimization of existing protocols for the isolation of diatom thylakoids

As outlined in the introduction, the literature provides protocols for the preparation of photosynthetically active thylakoid membranes or intact plastids from diatoms. Different methods have been used to break the silica frustule surrounding the diatom cell. Wittpoth et al. [55] have used a Yeda Press, which has the advantage that it can operate at very low pressures concomitant with lower shearing forces on the cells, membranes and protein complexes (1.5 bar, approx. 22 psi in the method of [55]), thus avoiding damage of subcellular structures. The Yeda Press method has been successfully employed in the purification of active thylakoid membranes from *Odontella sinensis* and *Coscinodiscus granii*. The other methods that have been used are cell lysis by freeze-thaw cycles, sonication or breakage of the silica cell wall by a French Pressure cell. These methods are, in general, widely used in cell biology. One freeze-thaw cycle was used by Nagao et al. [39] for the isolation of oxygen evolving thylakoids from the diatom species *Chaetoceros gracilis*, while Martinson et al. [36] used either sonication or a French Pressure cell to prepare active thylakoid membranes from *Cylindrotheca fusiformis*. However, as stated by Martinson et al. [36], freezing the diatom cells could lead to a loss of photosynthetic electron transport activity. In contrast to the results obtained by Nagao et al. [39], we were unable to break *C. meneghiniana* cells by one freeze-thaw cycle, as tested in preceding experiments. We therefore had to rely on the French Pressure cell to break the silica cell wall. However, to avoid damage to the thylakoid membranes during cell lysis we chose to operate the French Pressure cell at the very low pressure of 1.000 psi. This pressure was significantly lower compared to earlier methods used by us for thylakoid preparations from *P. tricornutum* or *C. meneghiniana* (see e.g. [32] where a pressure of 12.000 psi was used). Cell disruption using Pressure cells depends on the application of shearing forces, which can distort membranes to the point of fragmentation [22]. In a further attempt to decrease shearing stress on the membranes, i.e. in addition to the use of very low pressures, PEG 4000 was included in the isolation buffer. This polymer has been shown to stabilize chloroplasts of red and green algae during a purification procedure [37] or isolated thylakoid membranes during a temperature treatment [42]. Furthermore, PEG increases the viscosity of the isolation buffer, which likely reduces the shearing forces during the passage through the French Press. It may also decrease the diffusion of potentially harmful substances like, e.g., aldehydes and their oxidation products [31]. In contrast to the discontinuous sorbitol gradients proposed by Martinson et al. [36], where we observed an aggregation of isolated *C. meneghiniana* thylakoid membranes between the density interphases, the linear gradients used in the present study did not lead to a comparable aggregation during centrifugation.

### Purity of the isolated thylakoid membrane fraction

With respect to the purity of isolated diatom thylakoids the possible contamination with mitochondrial material is the most critical point, since plastids and mitochondria are characterized by similar centrifugation parameters. This can, for example, be seen by their close vicinity in a 2D Svedberg-density plot of cellular components [44]. In diatoms the separation of chloroplasts and mitochondria is even more complicated because the two organelles are tightly associated. A recent study using SEM-microscopy [12] has shown that in the pennate diatom *P. tricornutum* the mitochondrion appears as a continuous network which is directly attached to the envelope membrane of the chloroplast. In addition, it has been demonstrated that the physical contacts between the mitochondrion and the plastid serve to facilitate the energy exchange between the two organelles [2]. Depending on the stability of the chloroplast/mitochondrion association and the extent of these structures in centric diatoms like *C. meneghiniana* (for TEM images see e.g. [3, 45]), a more or less significant mitochondrial contamination of the thylakoids may occur during the preparation process.

**Fig. 2:**
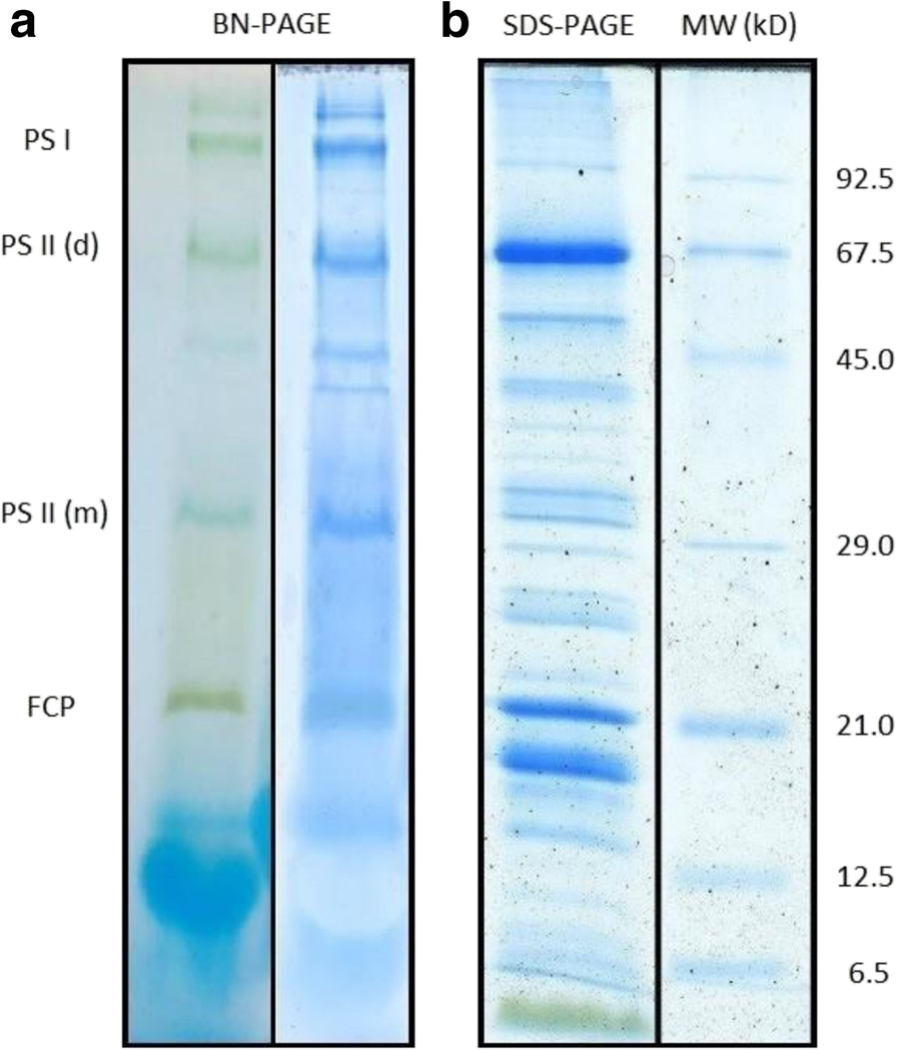
Protein composition of the purified thylakoid membranes determined by (**a**) blue-native (BN)-PAGE (left lane: unstained sample, right lane: coomassie stained) and (**b**) SDS-PAGE. For the BN-PAGE thylakoids were solubilised with a Chl concentration of 1 μg μL^−1^ and 8 μL were applied to the gel, for SDS-PAGE thylakoids were applied with a total Chl content of 1.5 μg to the gel. For additional information see the Methods section. PSI: photosystem I, PSII: photosystem II (d: dimeric state, m: monomeric state), FCP: fucoxanthin-chlorophyll binding protein, MW: molecular weight markers

One strategy for the purification of a specific membrane type from a cell lysate is the application of differential centrifugation, which uses centrifugation steps with different centrifugation times and centrifugal forces to pellet the various cell fractions. A simple two-step centrifugation for the isolation of diatom thylakoids for proteome research was described by Grouneva et al. [19]. In the respective study a pre-purified French Press extract was centrifuged at 20.000 g to isolate the thylakoid membrane fraction because the authors observed stronger contamination with mitochondria when thylakoids were harvested with 40.000 g. However, the obtained thylakoid fraction was contaminated with mitochondrial material. Increasing the purity of thylakoid fractions by differential centrifugation requires a series of centrifugation steps and is thus a time-consuming process, including the potential for membrane damage, because various steps of pellet resuspension and membrane aggregation are inevitable during the procedure. Therefore, for the present isolation protocol a separation of the French Press extract by a one-step centrifugation on a linear sorbitol density gradient was chosen. This technique makes use of the different density properties and centrifugal behaviour of the cellular components and membranes which depend on the differences of their physicochemical properties [44]. Linear sorbitol gradients from 2 to 3.3 M sorbitol were used for the present isolation, because in discontinuous sorbitol gradients, as used by Martinson et al. [36], we observed an unwanted aggregation of the isolated *C. meneghiniana* thylakoid membranes between the density interphases. To decrease the potential of mitochondrial contamination, the present gradient started with a high sorbitol concentration and thus a high density. In addition, centrifugal forces of only 16.000 g were chosen for the centrifugation step. Bright field and fluorescence microscopic images (Axioskop, Zeiss, Germany) of the isolated thylakoid fractions are depicted in Fig. 1b. They reveal a homogenous sample composition of green, spherical particles, which emit chlorophyll fluorescence upon excitation with blue light (HBO 100 W/2 with filter 470/40, Zeiss, Germany). Additional non-fluorescent particles, which would represent diatom mitochondria, were not observed or occurred in only very low amounts. Furthermore, the potential contamination of the thylakoid membrane fraction with mitochondria was analysed by blue-native (BN)-PAGE of the solubilised thylakoids. The band pattern of the BN-gel was clearly dominated by the pigment protein complexes of the thylakoid membrane (Fig. 2a). The separated pigment protein complexes included PSI, the PSII core complex in different oligomerization states and the main FCP. The pattern of the photosynthetic pigment proteins was comparable to those obtained by clear-native PAGE for *C. meneghiniana* and other diatoms by Nagao et al. [41]. Additional staining of the present gels with colloidal coomassie in order to identify potential mitochondrial protein complexes, i.e. the four main protein complexes of the respiratory electron transport chain, yielded only two additional minor bands. Since we used comparable conditions for the BN-PAGE as Perez et al. [43] for the separation of the complexes of the mitochondrial electron transport chain, these complexes would have become visible as prominent bands especially in the high molecular weight region of the BN-gel. Analysis by SDS-PAGE (Fig. 2b) showed a protein pattern dominated by the FCP bands which, for *C. meneghiniana*, are located at around 21 and 18 kDa [41]. The PSII reaction centre proteins D1 and D2 were visible as bands in the 30 to 35 kDa range [40]. The inner antenna proteins of PSII, i.e. CP47 and CP43, could be assigned to the protein bands above and below the 45 kDa marker [40]. The central subunits of PSI, the PsaA and PsaB core proteins, were visible as bands in the high molecular weight range slightly below the 67.5 kDa marker, in agreement with their high molecular mass. Taken together, the microscopic images and the protein analysis by BN- and SDS-PAGE indicate a high purity of the isolated thylakoid membranes and no or only minor contamination with other cell organelles, i.e. mitochondria.

The purity of the isolated *C. meneghiniana* thylakoids was also assessed by oxygen evolution/consumption measurements (Additional file 1) comparable to the measurements employed by Perez et al. [43] for the tests of mitochondrial activity. In a series of experiments, the electron donor of the mitochondrial electron transport NADH (1 mM) was added to the isolated thylakoids in the dark. Irrespective of the presence or absence of NADH oxygen consumption could not be detected, which supports the findings of the protein analysis and microscopic imaging that the diatom thylakoids prepared according to the modified protocol are essentially free of mitochondria. To test for a contamination with intact cells, the thylakoids were illuminated with actinic light in the absence of artificial electron acceptors. The absence of oxygen evolution under these conditions argues for the absence of intact cells in the present thylakoid preparation.

### Spectroscopic properties of the thylakoid preparation

#### Absorption spectroscopy

**Fig. 3:**
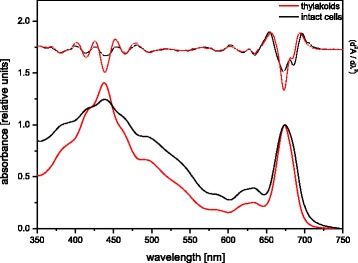
Absorption and second derivative spectra of intact cells and isolated thylakoid membranes of *C. meneghiniana*. The Chl concentration for the absorption measurements was 2 μg mL^−1^. For further measurement details see the Methods section. Figure. 3 shows representative spectra

Figure 3 shows absorption spectra of the isolated thylakoid membrane fraction in comparison to intact cells of *C. meneghiniana*. Both spectra are characterized by the typical pigment composition of diatoms with the Chl a Soret band at 438 nm and prominent absorption wavelengths for Chl c at 465 nm and 634 nm. Absorption by accessory antenna pigments was visible from 490 nm to 550 nm. While the absorption of the shorter wavelengths in this range was caused by fucoxanthin (Fx) and diadinoxanthin (DD), the long wavelength absorption up to 550 nm was most probably due to a special pool of Fx molecules [53]. The different ratios of the blue to red absorption maxima between isolated thylakoids and intact cells were due to differences in light scattering. A more particulate pigment system, like chloroplasts arranged in intact cells, induces a flattening effect in the blue part of the spectrum [8]. Differences in the wavelengths of the absorption maxima could be detected in the region of the Chl a Q_y_ band, localized between 650 nm and 700 nm. The thylakoid preparation resulted in a small hypsochromic shift to 673 nm for the Chl a maximum in the red part of the spectrum. Furthermore, the Chl a absorption peak at 674 nm was slightly broader in intact cells compared to the isolated thylakoids. These results indicate structural differences between the native thylakoid membranes within the intact chloroplast of *C. meneghiniana* and the isolated membranes after the preparation procedure. A further interesting effect in the Q_y_ region of the Chl a absorption was detected, which is related to changes of the two negative peaks that are visible in the second derivative of the cell and thylakoid spectra (Fig. 3). The shorter wavelength minimum localized at 672 nm may be related to PSII absorption because it was also detected in PSII fractions obtained after solubilisation of thylakoid membranes. PSI may be represented by the second absorption minimum at around 684 nm due to the longer wavelength absorbing Chl a molecules in PSI (for preparation details see [32]). Since the shorter wavelength peak at 672 nm was more pronounced after the thylakoid isolation, this may indicate that during the preparation PSII enriched membranes become more exposed. According to a recent model for the structure of diatom thylakoids, PSII is enriched in the inner lamellae within the stacks of the three thylakoid membranes, whereas PSI is mainly located in the outer lamellae ([33], see also [5]). Therefore, the observed shift towards an increased PSII absorption may indicate a destacking of the thylakoid membrane system, thereby exposing PSII enriched membrane sheets that are normally “buried” within the stacks *in vivo*.

#### 77 K fluorescence spectroscopy

**Fig. 4:**
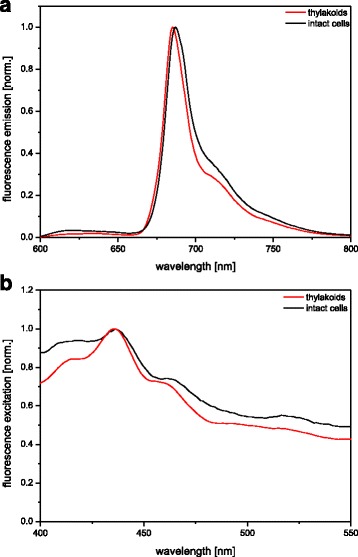
77 K fluorescence emission (**a**) and excitation spectra (**b**) of intact cells and isolated thylakoid membranes of *C. meneghiniana*. The Chl concentration for the 77 K fluorescence measurements was 1 μg mL^−1^. For additional measurement details see the Methods section. Figure 4 shows representative spectra

Structural differences between the intact cells and isolated thylakoids were also observed in the 77 K fluorescence spectra depicted in Fig. 4. Both emission spectra (Fig. 4a) showed Chl a fluorescence emission with a maximum located at around 685 nm and a prominent red shifted shoulder at around 715 nm, emitted by the antenna systems of PSII and PSI, respectively. The PSII fluorescence emission exhibited a broader peak in intact cells, due to a weakly pronounced additional emission shoulder located at 695 nm, which is most probably caused by the emission of the PSII core complexes. The isolated thylakoids were characterized by a small hypsochromic shift of the emission maximum to 685 nm (687 nm in intact cells), accompanied by a decrease of the fluorescence emission of the PSI antenna at around 715 nm and the PSII core at 695 nm. Differences of the fluorescence properties of isolated thylakoids compared with intact diatom cells were also reported in previous studies [36, 38] and were mainly attributed to mechanical stress that is unavoidably during the thylakoid preparation. The excitation spectra of the dominant emission peaks depicted in Fig. 4b were qualitatively comparable between intact cells and purified thylakoid membranes. Minor differences in the excitation intensity were most probably again induced by differences in light scattering and package effects, as described in the context of the presentation of the absorption spectra. In both excitation spectra, the most substantial contribution to the fluorescence emission was made by Chl a with an excitation maximum of 435 nm. Furthermore, in both spectra Chl c excitation was visible as a shoulder at around 460 nm. Excitation energy transfer from the carotenoids to Chl a was responsible for the excitation above 490 nm with a distinct shoulder at around 520 nm, which could be attributed to the long wavelength absorbing main light-harvesting pigment Fx. Taken together, the fluorescence emission and excitation properties of the isolated thylakoids indicate some differences in the excitation energy transfer to and within the two photosystems, most likely caused by the isolation procedure. Since fluorescence emission of free Chl a or Chl c molecules was not observed in the emission spectra of the thylakoid sample, it can be concluded that the pigment-protein complexes were preserved in a functional state.

### Electron transport characteristics of the thylakoid membranes

#### PSII and linear electron transport

The results from the oxygen evolution measurements (Table 1) showed that the *C. meneghiniana* thylakoids isolated with the present protocol retained a large part of their electron transport capacity in comparison to the intact cells. For the Hill reaction either DCBQ or FeCN were used as electron acceptors, the membranes were uncoupled with NH_4_Cl. DCBQ is an artificial quinone that penetrates into the hydrophobic phase of the thylakoid membrane and directly accepts electrons from PSII at the Q_B_ binding pocket. It is thus used to assess the PSII specific electron transport. In the presence of DCBQ the isolated thylakoids were able to produce around 117 μmol O_2_ mg Chl^−1^ h^−1^ upon illumination with a light intensity of 300 μmol m^−2^ s^−1^. Although this value is higher than rates reported by Martinson et al. [36] for *C. fusiformis*, a direct comparison of chlorophyll based oxygen yields is difficult for different diatom species and therefore probably not a direct indicator for the quality of the respective thylakoid preparation. Different diatom species show strong differences in the composition of their photosynthetic apparatus, which can lead to differences in the total amount of PSII and to different ratios of PSII to the other protein complexes of the photosynthetic electron transport chain [52]. This could also explain the high oxygen production rates obtained by Nagao et al. [39] for the marine diatom *C. gracilis*. Diatoms found in the marine habitat are often characterized by a high PSII/PSI ratio, which can be seen as an adaptation to the low iron abundance in the oceanic ecosystems [52]. In contrast to the quinone like acceptor DCBQ, the ionic electron acceptor FeCN is a polar molecule and not able to penetrate into the membrane. It is thus preferentially reduced by intact PSI. FeCN is also not able to pass through the chloroplast envelope and can only be applied in measurements with isolated thylakoids. The oxygen yield measured with FeCN represents the whole chain electron transport from the water-splitting site at PSII to the acceptor site of PSI. It is a measure of the integrity and intactness of all redox components of the complete electron transport chain. As shown in Table 1, oxygen yields with FeCN as acceptor were lower compared to the rates obtained with DCBQ and only reached values of around 30 μmol O_2_ mg Chl^−1^ h^−1^. Oxygen evolution with FeCN as electron acceptor was not only determined at a light intensity of 300 μmol photons m^−2^ s^−1^ but also at saturating light intensities which led to a further increase of the oxygen evolution rate (data not shown). However, to maintain the comparability of the present results with data from the literature [36], the main body of the experiments was done with the lower light intensity of 300 μmol m^−2^ s^−1^. Applying an osmotic shock to the isolated thylakoids did not increase the oxygen production rates, as it is typically observed when intact chloroplasts of higher plants are osmotically broken [34]. This indicates that the thylakoid fraction of the sorbitol gradient used in the present experiments was largely free of intact chloroplasts. Omission of the uncoupler NH_4_Cl decreased the oxygen evolution measured with either FeCN or DCBQ. The effect was more pronounced when FeCN was used as electron acceptor. In this case a decrease of the oxygen evolution, and thus electron transport, of around 20% in the untreated and around 30% in the shocked thylakoid membranes was observed (Table 1). This indicates that the isolated thylakoids were able to generate a light-driven proton gradient which, in the absence of uncoupler, exerted a certain inhibitory effect on the photosynthetic electron transport. However, this ∆pH was low compared to higher plant thylakoid preparations where addition of uncouplers normally increases the electron transport rates by a factor between three and four [27]. The ability of the thylakoid membranes to generate an, albeit low, proton gradient was supported by the 9-AAF quenching measurements presented below.

**Table 1:** Oxygen evolution of intact cells and isolated thylakoid membranes of *C. meneghiniana*. FeCN (2 mM) or DCBQ (0.5 mM) were used as electron acceptors, NH_4_Cl (10 mM) as uncoupler of the light-driven proton gradient. The Chl concentration of the cells or thylakoid membranes for the oxygen evolution measurements was 10 μg mL^−1^

Sample	Electron acceptor	Oxygen evolution [μmol O_2_ mg Chl^−1^ h^−1^]
Untreated thylakoids	DCBQ	117.44 ± 19.8
	FeCN; FeCN, −NH_4_Cl	29.48 ± 4.31; 23.43 ± 4.47
Shocked thylakoids	DCBQ	101.12 ± 9.84
	FeCN; FeCN, −NH_4_Cl	29.75 ± 6.7; 21.1 ± 1.02
Cells	No supplement	162.67 ± 28.99

For further information about the measurement conditions see the Methods section. Table 1 depicts the mean values of three independent thylakoid isolations with the respective standard deviations

#### Indication for chlororespiratory electron transport

The oxygen evolution measurements with DCBQ as electron acceptor resulted in an additional interesting observation. Additional file 1 shows a representative graph of the oxygen evolution measurements of the thylakoids. When DCBQ was used as electron acceptor a decrease in the oxygen concentration was regularly observed after a few minutes of illuminating the sample. Since this effect was only present in experiments with the artificial quinone, it seems likely that it is coupled to the reduction of DCBQ during the actinic illumination. Reduced DCBQ is then most probably re-oxidized enzymatically, accompanied by a simultaneous reduction of oxygen. Such a reaction is common in diatom thylakoids, it is conducted by an alternative oxidase and termed chlororespiration (for a review see [15]). Further experiments are needed to get a more detailed view of the reaction steps, which are involved in the diatom chlororespiratory electron transport chain and thylakoids isolated with the present protocol may serve to perform these experiments.

### PSII activity of the thylakoids determined by fast fluorescence induction measurements

**Fig. 5:**
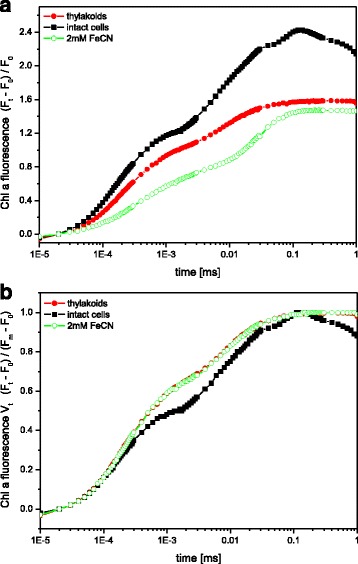
Fast fluorescence induction curves of intact cells and isolated thylakoids in the absence or presence of FeCN (2 mM) as electron acceptor. The induction curves were normalized to F_0_ (**a**) or F_0_ and F_m_ (**b**). The cells and thylakoids were measured with a Chl concentration of 2 μg mL^−1^. Figure 5 shows representative OJIP curves

Figure 5 depicts the fast fluorescence induction curves of intact cells and thylakoids. In contrast to the intact cells, primary photochemistry in isolated thylakoids seems to be modified by the preparation, probably in a similar manner as reported for isolated spinach thylakoids [54]. Fig. 5a, which depicts the fluorescence induction curves after normalization to F_0_, shows that the maximum fluorescence F_m_ is decreased in the isolated thylakoids compared to the intact cells. This is also reflected by the lower maximum quantum yield of PSII in isolated thylakoids (F_v_/F_m_ values of 0.61 in good preparations) in comparison to the intact cells (F_v_/F_m_ of 0.71). However, the F_v_/F_m_ values of the thylakoids show that the isolation procedure induced only a minor loss of PSII activity. A comparable pattern in the dynamic accumulation of reduced Q_A_ and Q_B_ for the cells and thylakoids can be seen in Fig. 5b, which shows the fluorescence induction curves after normalization to both F_0_ and F_m_. The double normalization corresponds to the relative variable fluorescence V_t_ (for a recent review see [51]). The comparable relative variable fluorescence of cells and thylakoids demonstrates that PSII is isolated in a functional state, as already concluded from the oxygen evolution measurements. The intact cells show a polyphasic rise of the fluorescence signal with a pronounced J-level, which is characterized by the presence of Q_A_^−^ Q_B_ and Q_A_^−^ Q_B_^−^ [21]. The I-level, which is normally attributed to the presence of Q_A_^−^ Q_B_H_2_, is not clearly defined in the fluorescence induction curve of the thylakoids. Intact cells are characterized by the presence of both the J- and the I-level. In comparison to the intact cells the fluorescence signal at the J-level is higher in the thylakoid membranes which indicates a faster reduction of Q_A_. Although this could be caused by the absence of electron acceptors, the OJIP curve of thylakoids in the presence of FeCN, which is comparable to thylakoids without artificial electron acceptors, argues against this scenario. It is thus likely that the thylakoid isolation led to a minor inhibition on the PSII acceptor side, resulting in the faster closure of the PSII reaction centre. Addition of DCBQ to isolated thylakoids resulted in a very low fluorescence signal and F_m_ could not be detected within the time span of the present measurements, i.e. 1 s. This is due to the fact that DCBQ represents a very efficient PSII electron acceptor, and thus keeps Q_A_ in a completely oxidized state during the time of the saturating flash used for the OJIP measurements. Comparable effects of DCBQ have also been observed in the higher plant *Pisum sativum* [7].

### NPQ and xanthophyll cycle activity of the isolated thylakoid membranes

**Fig. 6:**
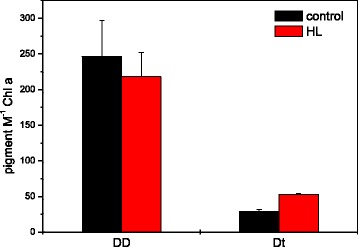
Diadinoxanthin (DD) and diatoxanthin (Dt) content of isolated thylakoids before (control) and after illumination with 1.000 μmol m^−2^ s^−1^ for 25 min in the presence of 30 mM ascorbate and 2 mM FeCN (HL). The illumination was performed at room temperature with a Chl concentration of 10 μg mL^−1^. DD and Dt concentrations are calculated as mM pigment M^−1^ Chl a. Fig. 6 shows the mean values of three independent thylakoid preparations with the respective standard deviations

The HPLC data (Fig. 6) showed that illumination of the isolated thylakoids with high light intensities led to a conversion of DD to Dt within the time-frame of 25 min that was used for the present measurements. However, the de-epoxidation reaction was not pronounced and only about 30 mM DD M^−1^ Chl a were de-epoxidized to Dt. This resulted in a change of the de-epoxidation state (DES) of the DD cycle pigment pool from 0.1 to 0.2 (data not shown). Although in the present thylakoid preparation a DD de-epoxidation could be detected, the efficiency of the conversion is low compared to intact *C. meneghiniana* cells where after a 10 min illumination with comparable light intensities a DES of almost 0.6 was determined [18]. Conversion of DD to Dt in the isolated diatom thylakoids was also significantly lower than the violaxanthin (V) de-epoxidation in spinach thylakoids where 30 min of actinic illumination with 800 μmol m^−2^ s^−1^ led to a DES of the V cycle pigment pool of 0.57 [14].

**Fig. 7:**
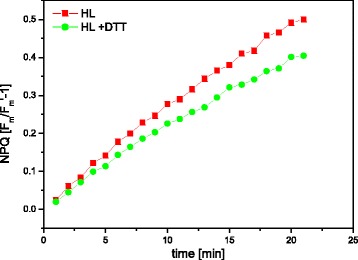
Time-course of NPQ of isolated thylakoids during illumination with 1.000 μmol m^−2^ s^−1^ for 25 min in the presence of 30 mM ascorbate and 2 mM FeCN (HL). 3 mM DTT was used to inhibit DD de-epoxidation (HL + DTT). The illumination was performed at room temperature with a Chl concentration of 10 μg mL^−1^. NPQ is calculated as (F_m_/F_m__′_-1). The measurements were done with three independent thylakoid preparations, Fig. 7 shows a representative measurement

The NPQ determination in the presence or absence of the de-epoxidation inhibitor DTT demonstrated that a small but detectable Dt-dependent NPQ was present in the isolated *C. meneghiniana* thylakoids (Fig. 7). In the presence of DTT NPQ was not as pronounced as in the thylakoids illuminated in the absence of DTT. In general, the NPQ values at the end of the 25 min illumination period with actinic high light were significantly lower than NPQ values reported for illuminated intact *C. meneghiniana* cells [18] or intact thylakoid membranes of spinach [14]. In the present measurements NPQ was dominated by photoinhibitory quenching qI, as demonstrated by the lack of fluorescence relaxation after the end of the actinic illumination period (data not shown). The low values of Dt-dependent NPQ are in line with the low amount of Dt after the high light illumination and the low DES. From the determination of DD cycle activity and NPQ it can be concluded that the thylakoids isolated according to the present preparation protocol have the potential for DD cycle and NPQ measurements. However, to systematically address these topics the preparation needs further refinement, e.g. lower pH-values during thylakoid preparation to retain higher concentrations of DD de-epoxidase [17]. Nevertheless, the present data show for the first time Dt-dependent NPQ and DD cycle activity in isolated diatom thylakoids. The inefficient DD de-epoxidation and NPQ are most probably caused by the small light-driven proton gradient established in the isolated thylakoids during actinic high light illumination (see below).

### Measurement of the ΔpH by 9-amino-acridine fluorescence quenching

**Fig. 8:**
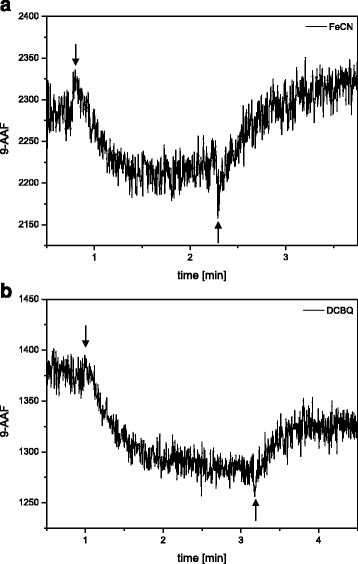
Original traces of 9-AAF quenching. Isolated thylakoids with a Chl concentration of 15 μg mL^−1^ were illuminated with a light intensity of 500 μmol m^−2^ s^−1^ in the presence of 2 mM FeCN (**a**) or 0.5 mM DCBQ (**b**). Arrows mark the onset and offset of the actinic illumination. 9-AA was used at a concentration of 6 μM. Figure 8 shows the traces of representative experiments

The 9-AAF measurements showed that the isolated thylakoid membranes were able to build up a light-driven proton gradient (Fig. 8). After the onset of actinic illumination, a decrease of the 9-AAF was observed in the presence of the artificial electron acceptors FeCN or DCBQ. The 9-AAF quenching reached its maximum after 1 to 2 min and a steady state was established. The induction time of 9-AAF quenching in the *C. meneghiniana* thylakoids corresponds well with kinetics for the build-up of the proton gradient in higher plant thylakoids where half-times of 1 to 1.5 min have been reported [13]. After switching off the actinic illumination the quenching of the 9-AAF showed a fast relaxation. Thus, the breakdown of the proton gradient exhibited comparable kinetics as the establishment of the ∆pH. Again, the relaxation kinetics are comparable to those documented for higher plants [13]. In the case of FeCN a slightly higher 9-AAF was observed after the relaxation of the proton gradient, whereas the use of DCBQ led to a slightly lower 9-AAF yield. The higher 9-AAF in the presence of FeCN can be explained by the overlap of FeCN absorption and 9-AAF excitation and emission, which can also be used for the simultaneous determination of the ∆pH and photosynthetic electron transport [11]. Reduction of FeCN during the actinic illumination used to drive the electron transport and the generation of the proton gradient leads to a reduced absorption of FeCN which, in turn, enhances the 9-AAF emission. The decrease of 9-AAF after the illumination period in the presence of DCBQ, however, is still unclear but may also be related to a shift of the absorption of the reduced quinone. The ∆F/F values, which serve as a measure for the magnitude of the ∆pH, showed values between 0.05 and 0.06 and were comparable for measurements with either DCBQ or FeCN as electron acceptor. In general, the ∆F/F values are low compared to respective values of freshly prepared higher plant thylakoids illuminated with saturating light intensities [13]. This is in line with the oxygen evolution measurements in the presence and absence of NH_4_Cl, which also indicated the presence of only a low ∆pH. It argues for a reduced capacity of the isolated *C. meneginiana* thylakoids to build up a proton gradient caused by a certain leakiness of the membranes induced by the thylakoid preparation. As a control 9-AAF was also measured in intact *C. meneghiniana* cells. Since the 9-AA molecules are not able to penetrate the diatom cell wall only an extremely low 9-AAF quenching could be detected upon illumination of the cells with actinic high light. The ∆F/F values that were calculated for these measurements were around 0.01 (data not shown). The very minor 9-AAF quenching is most probably related to a small amount of cells that were broken during the concentration of the cells by centrifugation, in order to achieve comparable Chl concentrations as they were used for the measurements with isolated thylakoid membranes. With respect to the determination of the proton gradient in diatoms it has to be mentioned that measurements of the electrochromic shift can be used to determine the membrane potential and the proton motive force in intact cells or isolated thylakoids. This method was recently employed by Bailleul et al. [2] to demonstrate that the strong energetic coupling between mitochondria and chloroplasts represents the driving force of CO_2_-assimilation in diatoms.

## Conclusions

The protocol presented in this study allows the isolation of active thylakoid membranes from the silicified diatom *C. meneghiniana*. The thylakoids show high PSII electron transport rates and maximum quantum yields which are comparable to those of intact cells. Interestingly, in addition to PSII and whole chain electron transport, indications for the presence of a chlororespiratory electron pathway were found. Spectroscopic data and results from fast fluorescence induction measurements demonstrate that the excitation energy transfer within the different pigment protein complexes and the intrinsic PSII electron transport are intact. The ability to build up a light-driven proton gradient, to de-epoxidize DD to Dt and to induce Dt-dependent NPQ has not been demonstrated before for diatom thylakoids. However, for detailed investigations of the xanthophyll cycle and NPQ the present isolation protocol has to be further optimized, in order to obtain thylakoid membranes that can induce a higher ∆pH. We conclude that thylakoid membranes prepared according to the present isolation protocol provide a good tool for studies concerning partial reactions of the linear or even chlororespiratory electron transport chain. In addition, the isolation of intact thylakoid membranes with a high oxygen evolution can be seen as an important step in the preparation and purification of oxygen-evolving PSII core complexes of *C. meneghiniana*.

## Additional file

Additional file 1: Original traces of oxygen evolution/consumption of the isolated thylakoids of *C. meneghiniana*. To test for the presence of intact cells in the thylakoid preparation the thylakoids were illuminated in the absence of artificial electron acceptors (black curve). To detect contamination with mitochondria the thylakoids were kept in the dark. NADH (1 mM) was then added as electron donor of the respiratory electron transport chain (green curve). Measurements were performed at room temperature, the thylakoid concentration was 10 μg Chl mL^−1^, the actinic light intensity was 300 μmol m^−2^ s^−1^. Arrows indicate different events, FeCN and DCBQ were added at final concentrations similar to oxygen evolution measurements. Additional file 2 Original traces of oxygen evolution/consumption during actinic illumination (300 μmol m^−2^ s^−1^) of the isolated thylakoids of *C. meneghiniana* in the presence of DCBQ (0.5 mM) or FeCN (2 mM). The uncoupler NH_4_Cl was used at a concentration of 10 mM. Thylakoids were used with a Chl concentration of 10 μg mL^−1^. (PDF 125 kb)

## Abbreviations

9-AAF: 9-amino-acridine fluorescence
BN-PAGE: Blue native polyacrylamide gel electrophoresis
Chl: Chlorophyll
DCBQ: 2,6-dichloro-*p*-benzoquinone
DD: Diadinoxanthin
DES: De-epoxidation state of the DD/Dt cycle pool
Dt: Diatoxanthin
DTT: Dithiothreitol
F_0_: Minimum PSII fluorescence
FCP: Fucoxanthin chlorophyll protein
FeCN: Ferricyanide
F_m_: Maximum PSII fluorescence
F_m'_: Maximum PSII fluorescence during actinic illumination
F_v_: Variable PSII fluorescence
Fx: Fucoxanthin
NPQ: Non-photochemical quenching of chlorophyll a fluorescence
PEG: Polyethylene glycole
PSI: Photosystem I
PSII: Photosystem II
Q_A_: Plastoquinone A
Q_B_: Plastoquinone B
SDS-PAGE: Sodium dodecyl sulfate polyacrylamide gel electrophoresis
V_t_: Relative variable fluorescence
