# Supplementary figures and images for: An optimized protocol for the preparation of oxygen-evolving thylakoid membranes from Cyclotella meneghiniana provides a tool for the investigation of diatom plastidic electron transport
Source: BMC Plant Biol. 2017 Nov 25;17:221. doi: 10.1186/s12870-017-1154-8 (PMC5702237; doi:10.1186/s12870-017-1154-8)

Additional file 1

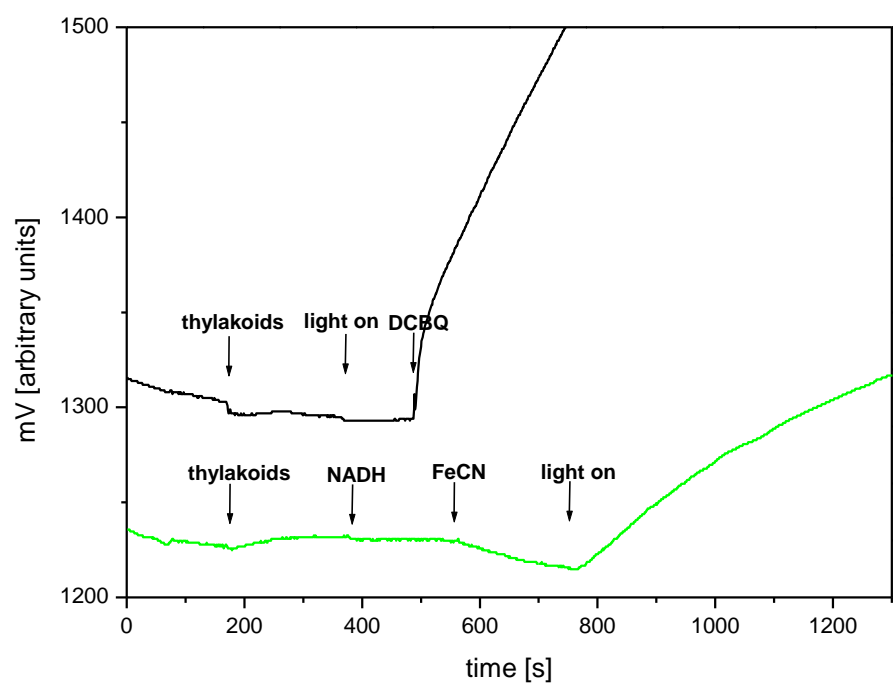

Additional file 2

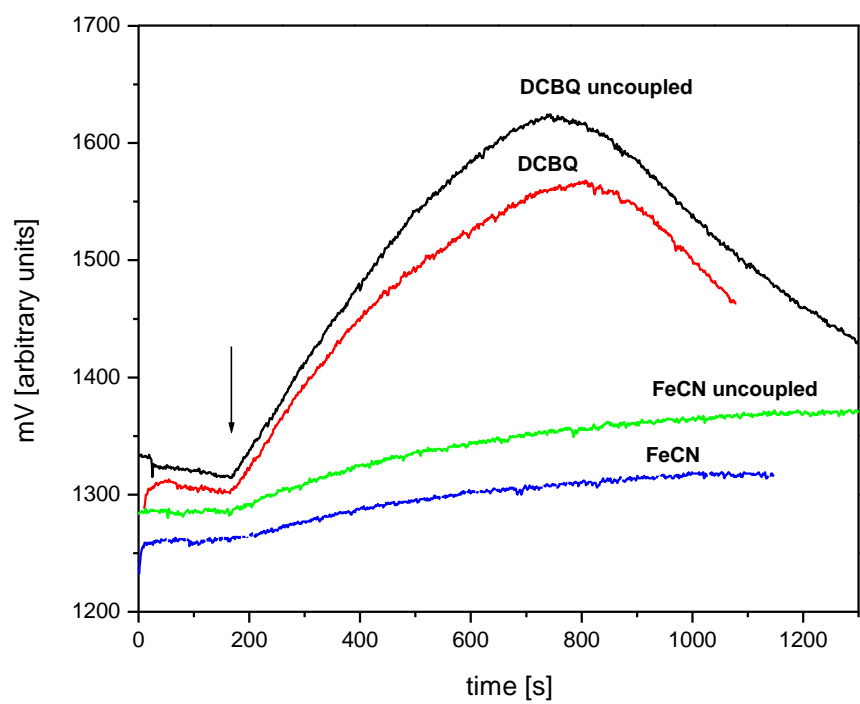

Supplement: Additional file 1: — Original traces of oxygen evolution/consumption of the isolated thylakoids of C. meneghiniana. To test for the presence of intact cells in the thylakoid preparation the thylakoids were illuminated in the absence of artificial electron acceptors (black curve). To detect contamination with mitochondria the thylakoids were kept in the dark. NADH (1 mM) was then added as electron donor of the respiratory electron transport chain (green curve). Measurements were performed at room temperature, the thylakoid concentration was 10 μg Chl mL−1, the actinic light intensity was 300 μmol m−2 s−1. Arrows indicate different events, FeCN and DCBQ were added at final concentrations similar to oxygen evolution measurements. Additional file 2 Original traces of oxygen evolution/consumption during actinic illumination (300 μmol m−2 s−1) of the isolated thylakoids of C. meneghiniana in the presence of DCBQ (0.5 mM) or FeCN (2 mM). The uncoupler NH4Cl was used at a concentration of 10 mM. Thylakoids were used with a Chl concentration of 10 μg mL−1. (PDF 125 kb) [file 12870_2017_1154_MOESM1_ESM.pdf]
